# Supplementary material for: Adiponectin-expressing Treg facilitate T lymphocyte development in thymic nurse cell complexes
Source: Commun Biol. 2021 Mar 16;4:344. doi: 10.1038/s42003-021-01877-w (PMC7966800; doi:10.1038/s42003-021-01877-w)

Supplementary Information

Supplementary Table 1. List of primers used for genotyping PCR.

Animals	Primer sequences	Products
Mice with *ADIPOQ* alleles (WT)	(F) 5'-CCAGAGAACAACGAACAAGGA-3'<br>(R) 5'-CGAATGGGTACATTGGGAAC-3'	473bp
Mice without *ADIPOQ* alleles (AKO)	(F) 5'-TGAATGAACTGCAGGACGAG-3'<br>(R) 5'-ATACTTTCTCGGCAGGAGCA-3'	171bp
Adn-CKO mice	(F) 5'- GTAGGTGATGTTCTGATGTGACCT-3'<br>(R) 5'- CATATCTTCGGGGCTGAGAGTGTT-3'	311bp (wild type)<br>416bp (mutant)
MMTV-PyVT mice	(F) 5'-GGAAGCAAGTACTTCACAAGGG-3'<br>(R) 5'-GGAAAGTCACTAGGAGCAGGG-3'	556bp
Adn-Cre mice	(F) 5'-GGATGTGCCATGTGAGTCTG-3'<br>(R) 5'-ACGGACAGAAGCATTTTCCA-3'	200bp
ROSA$^{mT/mG}$ mice	Common 5'-CTTTAAGCCTGCCCAGAAGA-3'<br>Mutant (F) 5'-TAGAGCTTGCGGAACCCTTC-3'<br>Wild type (F) 5'-AGGGAGCTGCAGTGGAGTAG-3'	212bp (wild type)<br>128bp (mutant)

Supplementary Table 2. List of primers used for RT-PCR or QPCR.

Gene	Experiment	Primer sequences	Products
ADIPOQ	RT-PCR	(F) 5’-AAGCTCTCCTGTTCCTCTTAATC-3’<br>(R) 5’-CATGGTAGAGAAGAAAGCCAGTA-3’	720bp
ADIPOQ	RT-PCR	(F) 5’-TGCCGAAGATGACGTTACTAC-3’<br>(R) 5’-GTAGAGTCGTTGACGTTATCT-3’	660bp
ADIPOQ	QPCR	(F) 5’-CTCCACCCAAGGGAACTTGT-3’<br>(R) 5’-TAGGACCAAGAAGACCTGCATC-3’	141bp
FGF21	QPCR	(F) 5'-TATGGATCGCCTCACTTTGA -3'<br>(R) 5'-GGAGTCCTTCTGAGGCAGAC -3'	123bp
PPARA	QPCR	(F) 5'-GAGTGCAGCCTCAGCCAAGTTGA -3'<br>(R) 5'-AGGCAGGCCACAGAGCGCTAA -3'	121bp
TNFA	QPCR	(F) 5'- TCGTAGCAAACCACCAAGTG-3'<br>(R) 5'- AGATAGCAAATCGGCTGACG -3'	207bp
CCL2	QPCR	(F) 5'-TGATCCCAATGAGTAGGCTGGAG -3'<br>(R) 5'-ATGTCTGGACCCATTCCTTCTTG -3'	132bp
TGFB1	QPCR	(F) 5'-TGGAGCAACATGTGGAACTC-3'<br>(R) 5'-CGTCAAAAGACAGCCACTCA -3'	108bp
IL6	QPCR	(F) 5'-TGGAGTCACAGAAGGAGTGGCTAAG-3<br>(R) 5'-TCTGACCACAGTGAGGAATGTCCAC-3'	155bp
VEGFA	QPCR	(F) 5'-AACGATGAAGCCCTGGAGTG-3'<br>(R) 5'-TGAGAGGTCTGGTTCCCGA-3'	120bp
RNA18S	QPCR	(F) 5'-AGTCCCTGCCCTTTGTACACA-3'<br>(R) 5'-CGATCCGAGGGCCTCACTA-3'	100bp
ACTB	QPCR	(F) 5'-AGTGTGACGTTGACATCCGT -3'<br>(R) 5'-CCACCGATCCACACAGAGTA -3'	178bp

Supplementary Table 3. List of antibodies for Western blotting (WB) or immunofluorescence (IF).

**Antibody (*species*)**	**Company**	**Catalog number**	**Dilution (application)**
Anti-murine adiponectin (*rabbit*)	Immunodiagnostics, Hong Kong, China	12010	1:3000 (WB), 1:1000 (IF)
Anti-cytokeratin 5 (*goat*)	Santa Cruz Biotechnology, Santa cruz, CA	sc-17090	1:50 (IF)
Anti-cytokeratin 8 (*goat*)	Santa Cruz Biotechnology, Santa cruz, CA	sc-241376	1:50 (IF)
Anti-neuropilin-1 (*goat*)	R&D system Inc., Minneapolis, MN, USA	AF566	5 μg/ml (IF)
Anti-CD31 (*goat*)	R&D system Inc., Minneapolis, MN, USA	AF3628	1:200 (IF)
Anti-galectin-3 (*rat*)	Santa Cruz Biotechnology, Santa cruz, CA	sc-23938	1:200 (WB), 1:100 (IF)
Anti-β5t (*rabbit*)	MBL Life science, Nagoya, Japan	PD021	1:200 (IF)
Anti-β-actin (*mouse*)	Sigma-Aldrich, St Louis, MI, USA	A1978	1:1000 (WB)
Anti-neuropilin-1 (*mouse*)	R&D, Minneapolis, MN, USA.	AF566	1:300 (IF)
Anti-CD72 (*mouse*)	R&D, Minneapolis, MN, USA.	AF1279	1:300 (IF)
Anti-CD100 (*mouse*)	Abcam, Cambridge, UK	Ab231961	1 μg/ml (WB), 5 μg/ml (IF)
Anti-CD100 (*human*)	LS-Bio, Seattle, WA, USA	LS-B12098-50	1:2000 (WB)
Anti-CD100 (*mouse*)	BD Biosciences	565556	1:100 (IF)

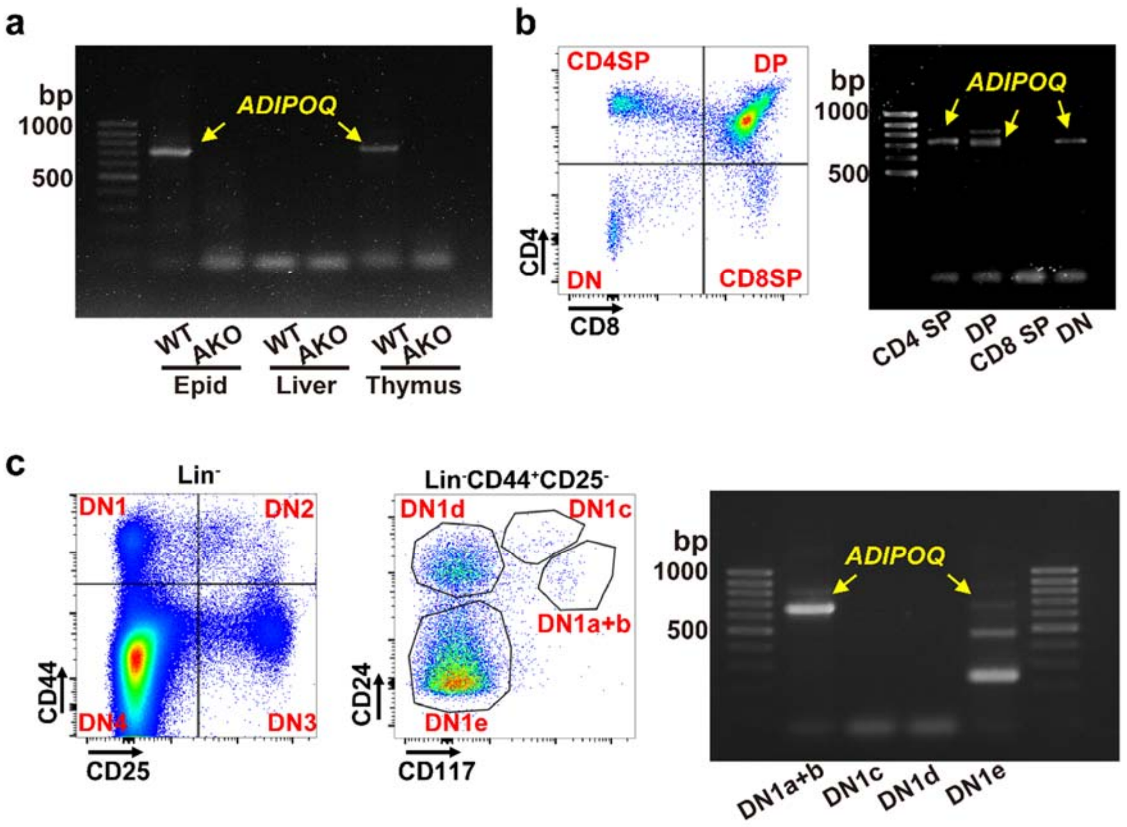

Supplementary Figure 1. Adiponectin expression in thymus. Mice were sacrificed at the age of seven-weeks for analyzing the mRNA expression of *ADIPOQ*. **a** RT-PCR was performed to detect the expression of full-length *ADIPOQ* transcript in epididymal adipose tissue [epid], liver and thymus collected from WT or AKO mice. **b** RT-PCR was performed to detect the expression of full-length *ADIPOQ* transcript in CD4 SP, DP, CD8 SP and DN populations of thymocytes isolated from WT mice. **c** Nested RT-PCR was performed to detect the expression of *ADIPOQ* transcript in DN1a+b, DN1c, DN1d and DN1e subpopulations of thymocytes isolated from WT mice.

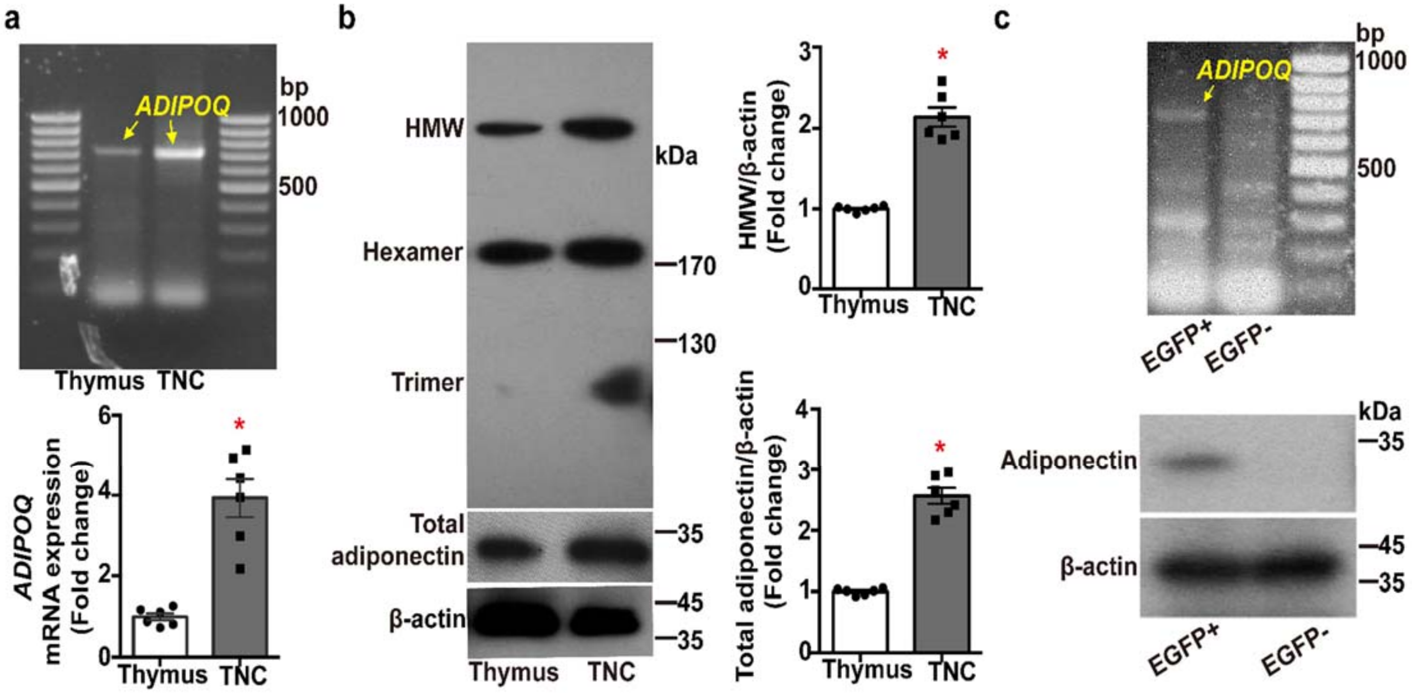

Supplementary Figure 2. Adiponectin expression in TNC complexes or EGFP$^+$ cells. a WT mice were sacrificed at the age of seven-weeks for analyzing the mRNA expression of *ADIPOQ* in thymus and enriched TNC complexes. RT-PCR was performed to detect the expression of full-length *ADIPOQ* transcript (*top*). QPCR was performed for measuring the relative mRNA expression levels of *ADIPOQ* (*bottom*). After normalization against 18S rRNA, results are presented as fold changes for comparison. **b** Thymus and TNC complexes were prepared from seven-weeks old WT mice. The oligomers and total protein amount of adiponectin were analyzed by non-reducing and denatured SDS-PAGE, respectively, followed by Western blotting (*left*). Beta-actin (β-actin) was probed as loading controls. The relative abundance of HMW and total adiponectin was quantified by calculating the ratios with β-actin, respectively, for comparison (*right*). **c** EGFP$^+$ and EGFP$^-$ thymocytes were isolated from Adn-Cre/ROSA$^{mT/mG}$ mice. RT-PCR was performed to detect the expression of full-length *ADIPOQ* transcript (*top*). Adiponectin protein expression was analyzed by denatured SDS-PAGE and detected by Western blotting (*bottom*). Data are presented as mean ± SEM. *, $P<0.05$ vs thymus (n=6 biologically independent samples of independent experiment).

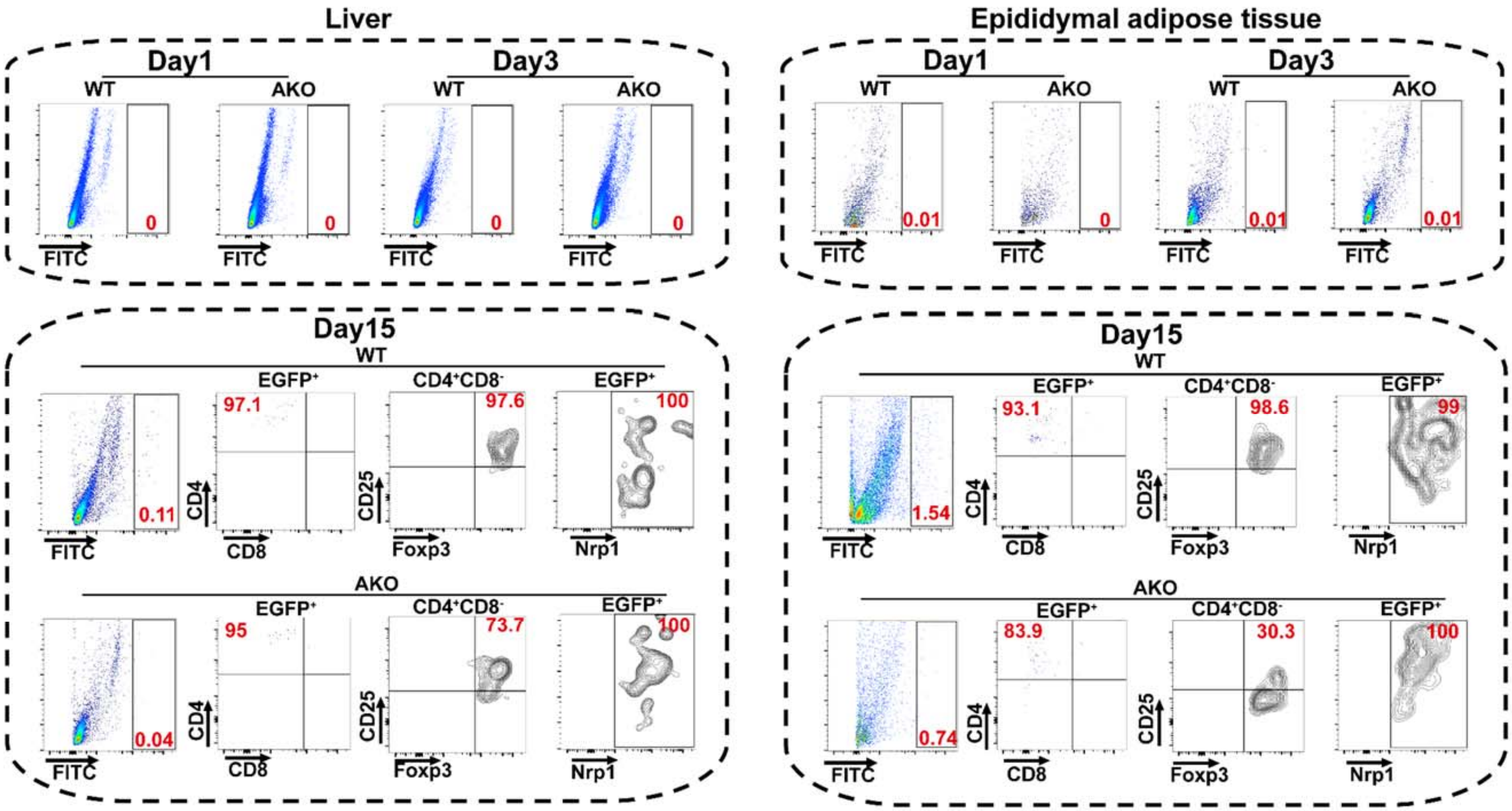

Supplementary Figure 3. The distribution of adiponectin-expressing Treg in liver and epididymal adipose tissue after adoptive transfer. Adiponectin-expressing $EGFP^{+}$ thymocytes collected from Adn-Cre/$ROSA^{mT/mG}$ were injected [30000 cells/*mouse* via tail vein] into WT or AKO mice. On the 1st, 3rd and 15th day after injection, lymphocytes were isolated from liver (left) and adipose tissue (right) of WT and AKO recipient mice, as described in Methods. Flow cytometry was performed to analyze the distribution of $EGFP^{+}$ cells after staining with antibodies recognizing CD4, CD8, CD25, Foxp3 and Nrp1.

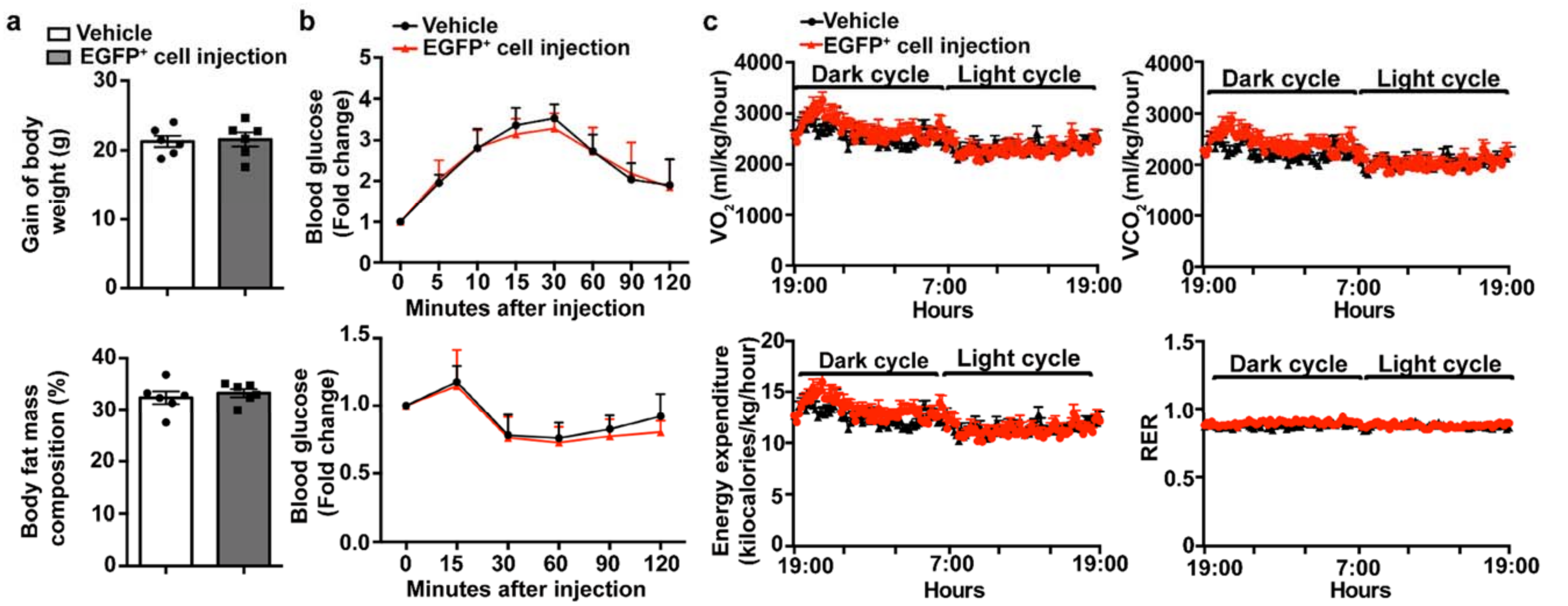

Supplementary Figure 4. Treatment with adiponectin-expressing tTreg precursors did not alleviate HFD-induced metabolic abnormalities in AKO mice. Vehicle or adiponectin-expressing $EGFP^{+}$ cells [30000 cells/*mouse*] isolated from Adn-Cre/$ROSA^{mT/mG}$ were injected via tail vein into four-weeks old AKO mice, which were then subjected to HFD feeding for another 12-weeks. **a** At the end of treatment, the gain of body weight and the percentage body fat mass composition were calculated for comparison. **b** After 10- and 12-weeks of HFD, intraperitoneal glucose (*top*) and insulin (*bottom*) tolerance tests were performed for comparison. **c** Indirect calorimetry was used to examine the VO_2, VCO_2, energy expenditure and RER for comparison. Data are presented as mean ± SEM (n=6 biologically independent animals of independent experiment).

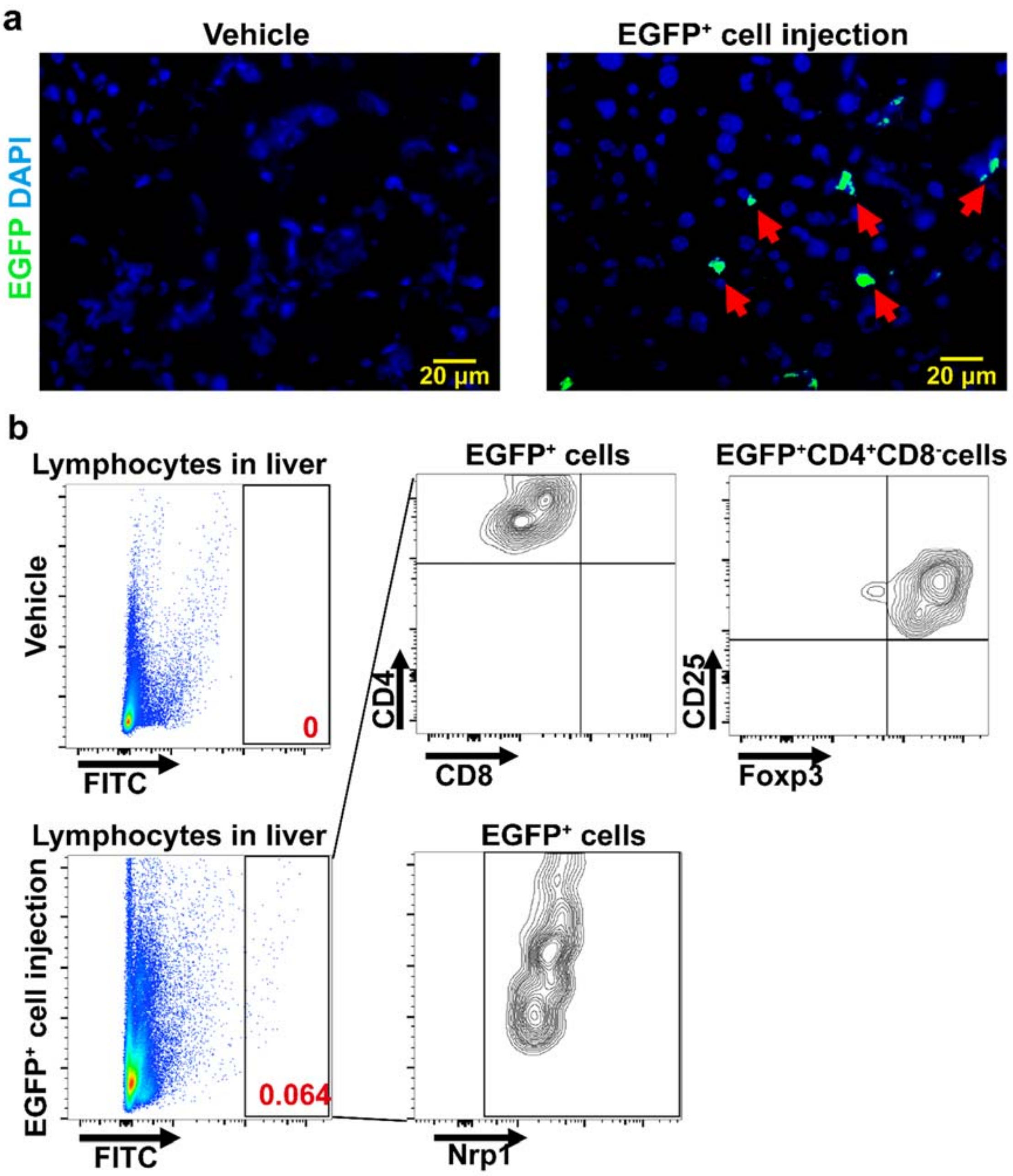

Supplementary Figure 5. Distribution of EGFP^{+} cells in liver after adoptive transfer in WT mice fed with HFD. Vehicle or adiponectin-expressing EGFP^{+} thymocytes [30000 cells/*mouse*] isolated from Adn-Cre/ROSA$^{mT/mG}$ were injected via tail vein into four-weeks old WT mice, which were then subjected to HFD as in Figure 5. **a** Frozen tissue sections were prepared from liver to visualize the EGFP^{+} cells. **b** Flow cytometry was performed to analyze the distribution of EGFP^{+} cells after staining with the markers, including CD4, CD8, CD25, Foxp3 and Nrp1.

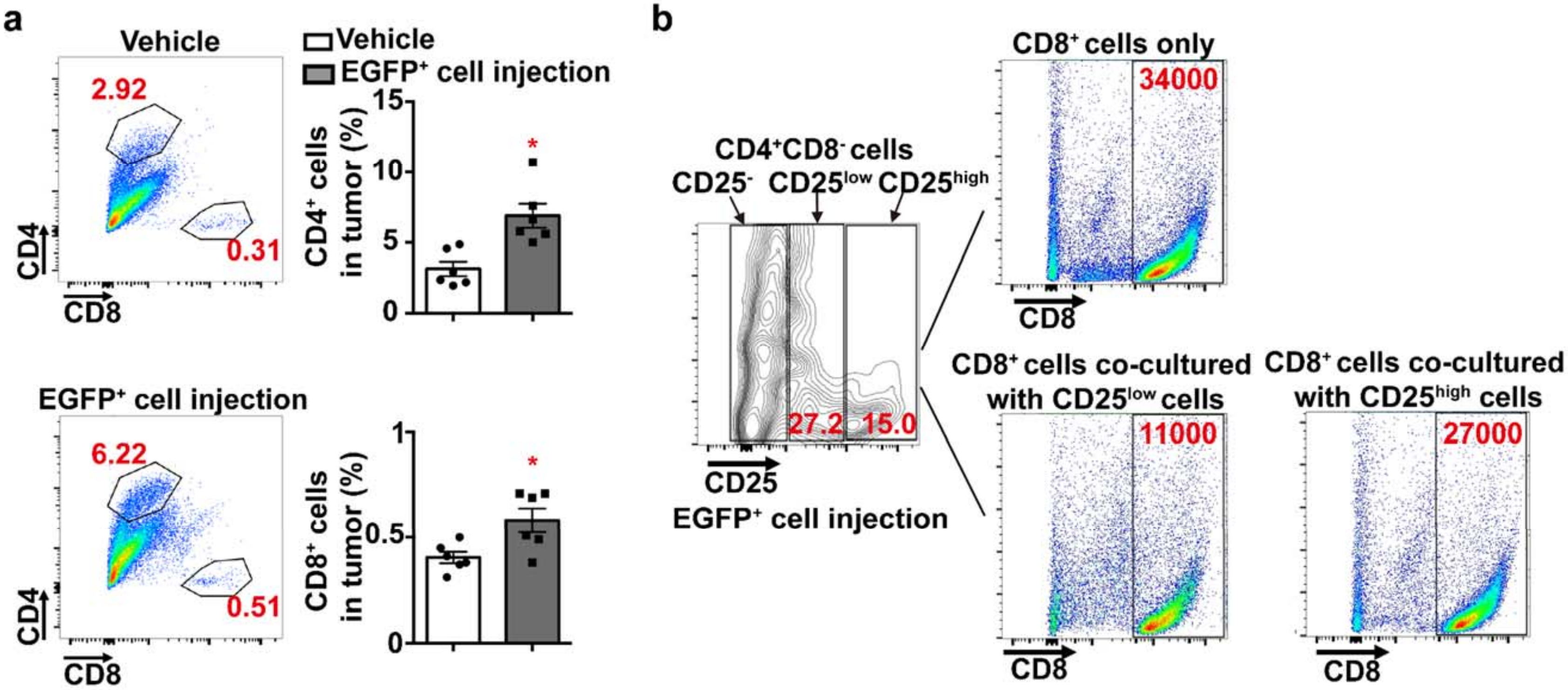

Supplementary Figure 6. Analyses of T lymphocytes in tumors from MMTV-PyVT mice treated with vehicle or EGFP$^+$ thymocytes. Adoptive transfer of adiponectin-expressing EGFP$^+$ thymocytes (30000 cells/*mouse* via tail vein) was performed in MMTV-PyVT mice at the age of four-weeks. Mammary tumors were collected at the age of 14-weeks for analyses. **a** The amount of CD4$^+$ and CD8$^+$ cells was examined by flow cytometry for comparison. **b** The CD4$^+$CD8$^-$CD25^{low} or CD4$^+$CD8$^-$CD25^{high} EGFP$^+$ cells ($1.25x10^4$) were isolated from mammary tumor tissues and mixed with $2.5x10^4$ of CD8$^+$ cells. After co-culturing in RPMI 1640 medium with 10% FBS for 72 hours, flow cytometry was performed to quantify the number of CD8$^+$ cells in each group. Data are shown as means ± SEM. *, P<0.05 vs vehicle controls (n=6 biologically independent samples from different animals of independent experiment).

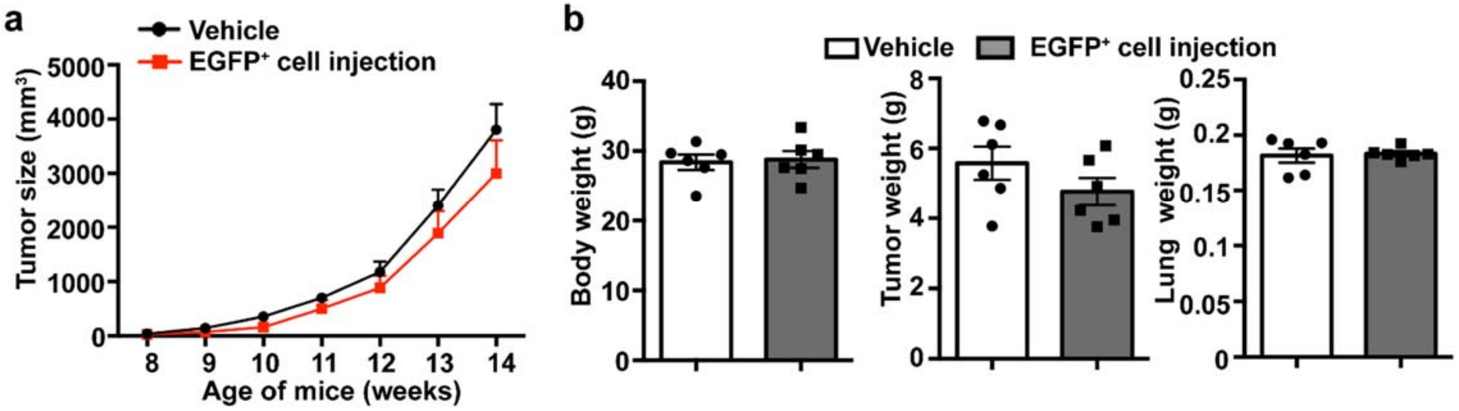

Supplementary Figure 7. Treatment with adiponectin-expressing tTreg precursors did not inhibit breast cancer development in PyVT-AKO mice. EGFP+ cells collected from the thymus of Adn-Cre/ROSA$^{mt/mg}$ mice were adoptively transferred into four-weeks old PyVT-AKO mice via tail vein injection [30000 cells/*mouse*]. **a** Mammary tumor development was monitored once per week as described in Methods for comparison. **b** At the age of 14-weeks, mice were sacrificed to measure the body, tumor and lung tissue weights for comparison. Data are shown as means ± SEM (n=6 biologically independent animals of independent experiment).

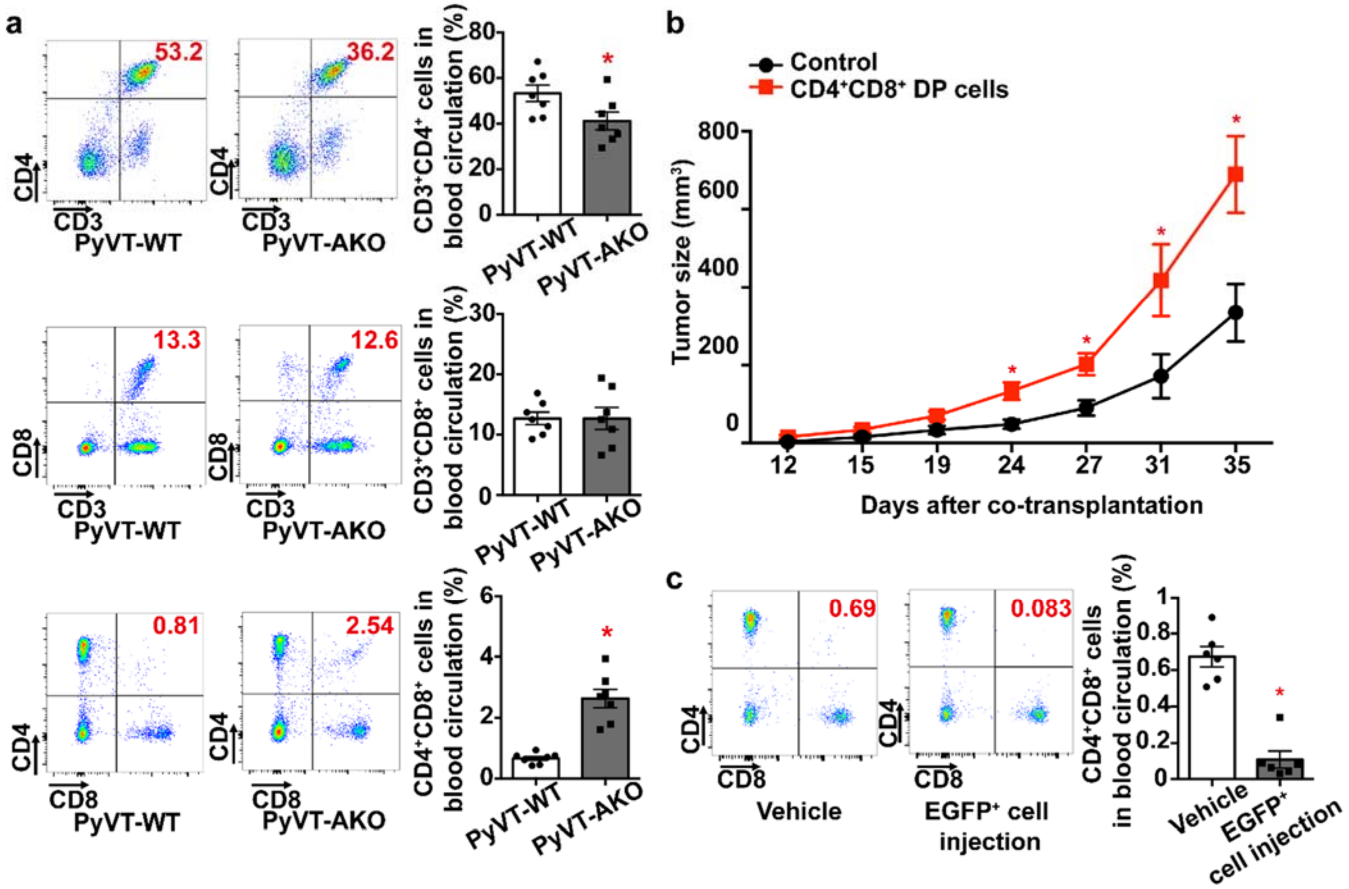

Supplementary Figure 8. Co-implantation of $CD4^{+}CD8^{+}$ cells promoted MBA-MB-231 tumor development. a PyVT-WT or PyVT-AKO mice were sacrificed at the age of seven-weeks. Flow cytometry was performed to analyze the populations of $CD3^{+}CD4^{+}$, $CD3^{+}CD8^{+}$ and $CD4^{+}CD8^{+}$ cells in the blood circulation for comparison. **b** The human breast cancer MDA-MB-231 cells (2×10^{5}) were implanted into the mammary fat pads of female NOD/SCID mice together without (Control) or with the $CD4^{+}CD8^{+}$ DP cells (3000 cells/*mouse*) isolated from the blood circulation. Tumor development was monitored for comparison. **c** Vehicle or adiponectin-expressing $EGFP^{+}$ thymocytes (30000 cells/*mouse*) were injected via tail vein into MMTV-PyVT mice at the age of four-weeks. Three-weeks later, flow cytometry was performed to analyze the populations of CD4+CD8+ cells in the blood circulation. Data are shown as means ± SEM. *, $P<0.05$ vs corresponding controls (n=6 biologically independent samples from different animals of independent experiment).

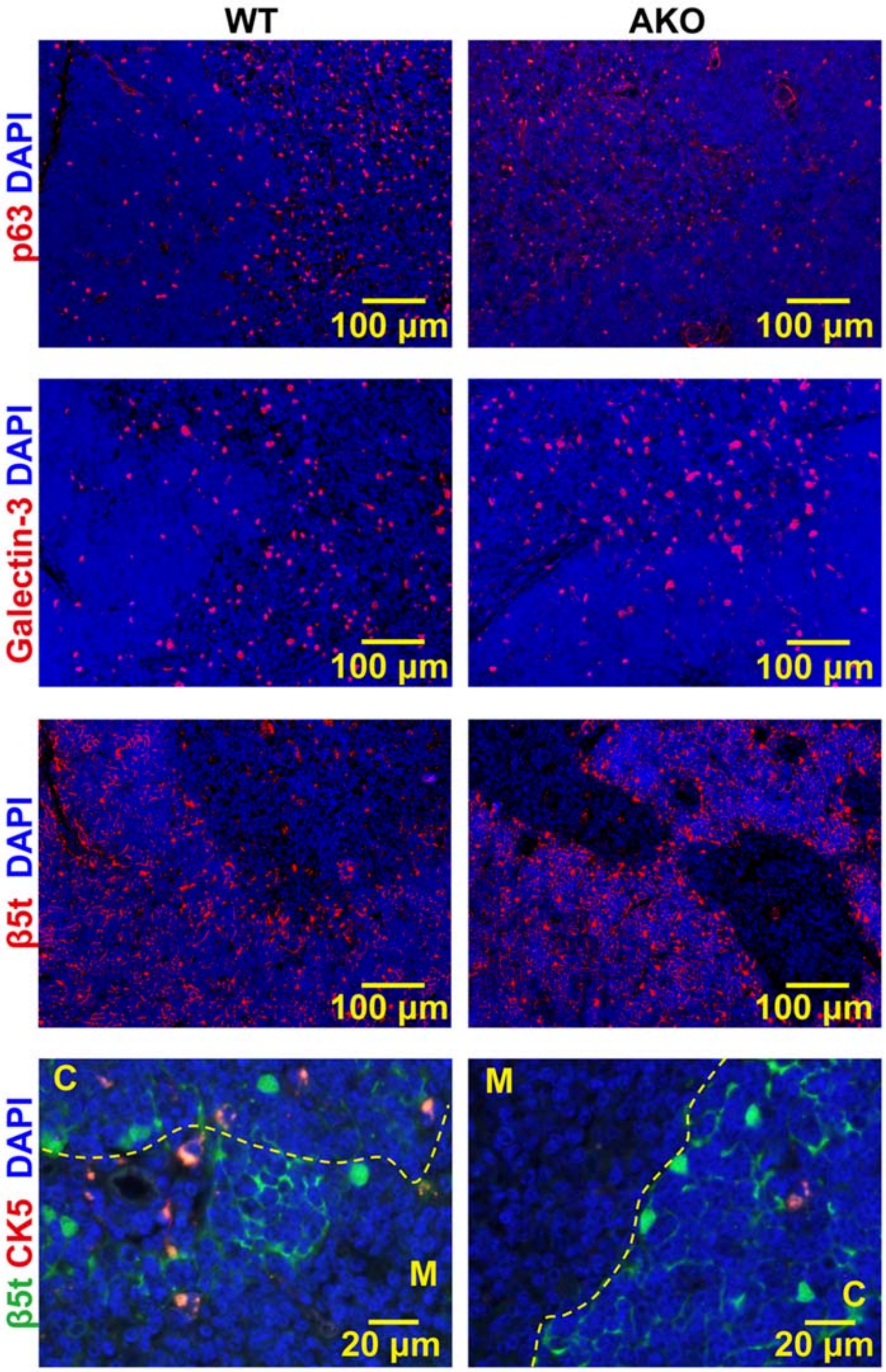

Supplementary Figure 9. Comparison of the epithelial microenvironment in thymus of WT or AKO mice. Thymus tissue sections were prepared from seven-weeks old WT and AKO mice. Immunofluorescence staining was performed to examine the expression and distribution of epithelial makers, including p63, galectin-3, β5t and cytokeratin 5 [CK5].

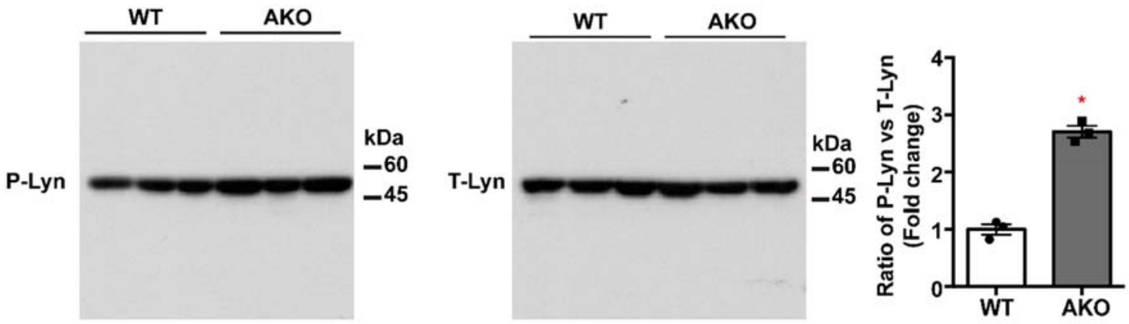

Supplementary Figure 10. Comparison of Lyn expression in thymus. Western blotting was performed to analyze the protein levels of phosphorylated Lyn (P-Lyn) in thymus prepared from seven-weeks old WT and AKO mice. Total protein expression of Lyn (T-Lyn) was measured for calculating the ratios between P-Lyn and T-Lyn for comparison. Fold changes were calculated for comparison. Data are presented as mean ± SEM. *, $P<0.05$ vs WT (n=3 biologically independent samples from different animals).

Gating strategy for flow cytometric analyses

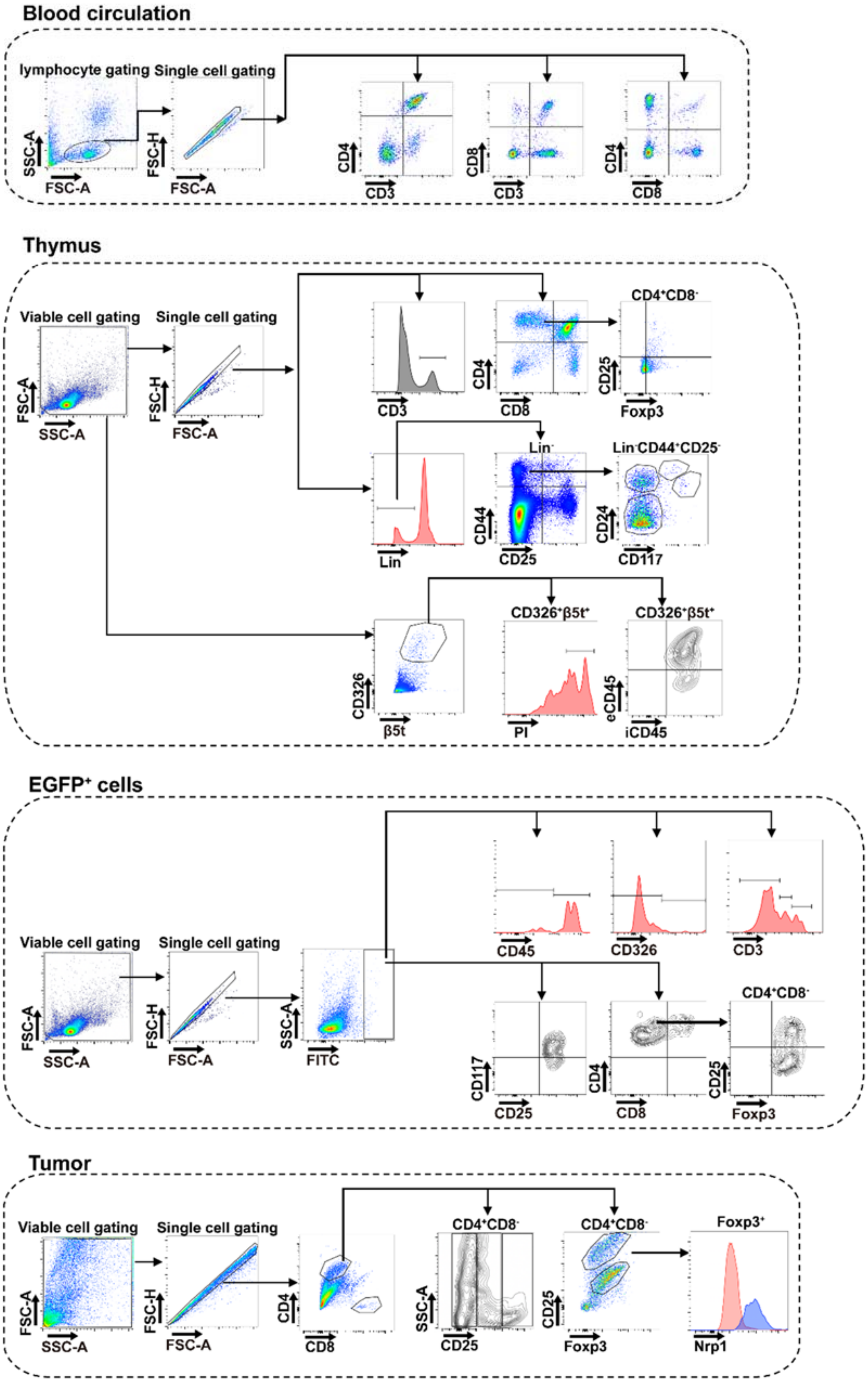

Full gel images of Western blotting results in Figure 9

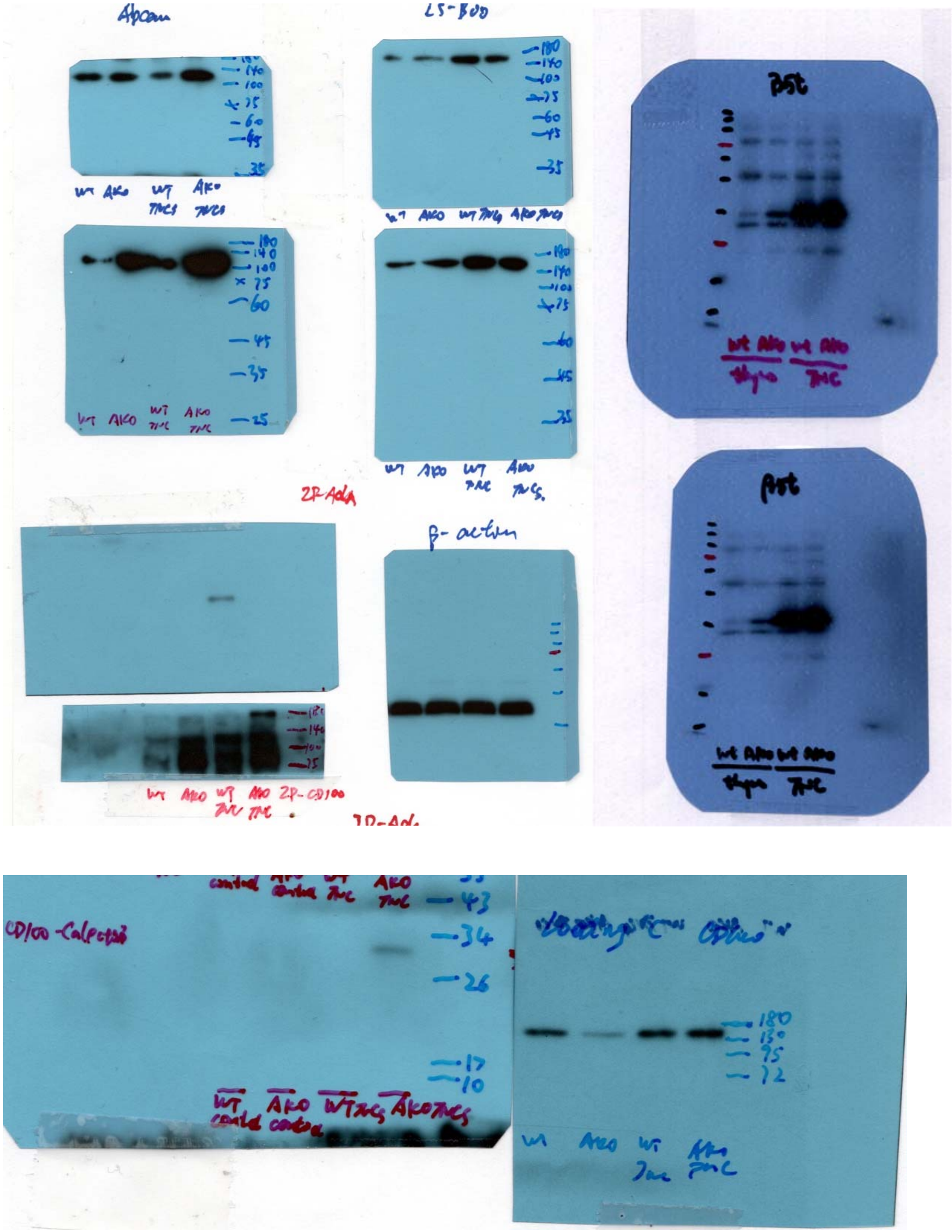

Supplement: Supplementary file 2 — Supplementary Information [file 42003_2021_1877_MOESM2_ESM.pdf]
