# Supplementary material for: Adiponectin-expressing Treg facilitate T lymphocyte development in thymic nurse cell complexes
Source: Commun Biol. 2021 Mar 16;4:344. doi: 10.1038/s42003-021-01877-w (PMC7966800; doi:10.1038/s42003-021-01877-w)
Supplement: Supplementary file 3 — Description of Additional Supplementary Files [file 42003_2021_1877_MOESM3_ESM.pdf]

Description of Additional Supplementary Files

File Name: Supplementary Movie 1

Description: Enriched TNC complexes were prepared from the thymus of Adn-Cre/ROSAmT/mG mice and then cultured in phenol red-free DMEM using a temperature (37℃) controlled-chamber. The 3D live cell images were acquired at a 15-minutes intervals using the UltraVIEW® VOX Spinning Disc confocal system (Perkin Elmer) equipped with a confocal fluorescent microscopy. DAPI (4′,6-diamidino-2-phenylindole; 20 μg/ml) was added into the chamber before image acquisition.

File Name: Supplementary Movie 2

Description: Enriched TNC complexes were prepared from the thymus of Adn-Cre/ROSAmT/mG-AKO mice and then cultured in phenol red-free DMEM using a temperature (37℃) controlled-chamber. The 3D live cell images were acquired at a 15-minutes intervals using the UltraVIEW® VOX Spinning Disc confocal system (Perkin Elmer) equipped with a confocal fluorescent microscopy. DAPI (4′,6-diamidino-2-phenylindole; 20 μg/ml) was added into the chamber before image acquisition.
