# Supplementary material for: Adiponectin-expressing Treg facilitate T lymphocyte development in thymic nurse cell complexes
Source: Commun Biol. 2021 Mar 16;4:344. doi: 10.1038/s42003-021-01877-w (PMC7966800; doi:10.1038/s42003-021-01877-w)
Supplement: Supplementary file 6 — Reporting Summary [file 42003_2021_1877_MOESM6_ESM.pdf]

nature research

Corresponding author(s): Yu Wang

Last updated by author(s): Jan 26, 2021

Reporting Summary

Nature Research wishes to improve the reproducibility of the work that we publish. This form provides structure for consistency and transparency in reporting. For further information on Nature Research policies, see our Editorial Policies and the Editorial Policy Checklist.

Statistics

For all statistical analyses, confirm that the following items are present in the figure legend, table legend, main text, or Methods section.

n/a	Confirmed	
☐	☒	The exact sample size (n) for each experimental group/condition, given as a discrete number and unit of measurement
☐	☒	A statement on whether measurements were taken from distinct samples or whether the same sample was measured repeatedly
☐	☒	The statistical test(s) used AND whether they are one- or two-sided<br>*Only common tests should be described solely by name; describe more complex techniques in the Methods section.*
☒	☐	A description of all covariates tested
☒	☐	A description of any assumptions or corrections, such as tests of normality and adjustment for multiple comparisons
☒	☐	A full description of the statistical parameters including central tendency (e.g. means) or other basic estimates (e.g. regression coefficient) AND variation (e.g. standard deviation) or associated estimates of uncertainty (e.g. confidence intervals)
☒	☐	For null hypothesis testing, the test statistic (e.g. F, t, r) with confidence intervals, effect sizes, degrees of freedom and P value noted<br>*Give P values as exact values whenever suitable.*
☒	☐	For Bayesian analysis, information on the choice of priors and Markov chain Monte Carlo settings
☒	☐	For hierarchical and complex designs, identification of the appropriate level for tests and full reporting of outcomes
☒	☐	Estimates of effect sizes (e.g. Cohen's d, Pearson's r), indicating how they were calculated

Our web collection on statistics for biologists contains articles on many of the points above.

Software and code

Policy information about availability of computer code

Data collection	Volocity (Version: 6.3.0, Perkin Elmer), BD FACSDiva™ (Version 6.1, BD), AxioVision Imaging Plus software (4.9.1, Carl Zeiss Ltd.)
Data analysis	Image J (Version, 1.51, NIH, U.S.A.), Volocity (Version: 6.3.0, Perkin Elmer), FlowJo (Version: 10.0.7, BD), AxioVision Imaging Plus software (4.9.1, Carl Zeiss Ltd.)

For manuscripts utilizing custom algorithms or software that are central to the research but not yet described in published literature, software must be made available to editors and reviewers. We strongly encourage code deposition in a community repository (e.g. GitHub). See the Nature Research guidelines for submitting code & software for further information.

Data

Policy information about availability of data

All manuscripts must include a data availability statement. This statement should provide the following information, where applicable:

- Accession codes, unique identifiers, or web links for publicly available datasets
- A list of figures that have associated raw data
- A description of any restrictions on data availability

We state that all figures have associated raw data and there are no restrictions on data availability.

Field-specific reporting

Please select the one below that is the best fit for your research. If you are not sure, read the appropriate sections before making your selection.

☒ Life sciences ☐ Behavioural & social sciences ☐ Ecological, evolutionary & environmental sciences

For a reference copy of the document with all sections, see nature.com/documents/nr-reporting-summary-flat.pdf

Life sciences study design

All studies must disclose on these points even when the disclosure is negative.

Sample size	For all experiments, the information of n number were included in the figure legends.
Data exclusions	No data exclusions
Replication	All experimental findings are reproducible. All attempts at replication were successful.
Randomization	Mice were allocated into experimental groups randomly.
Blinding	Blinding is not relevant to this study.

Reporting for specific materials, systems and methods

We require information from authors about some types of materials, experimental systems and methods used in many studies. Here, indicate whether each material, system or method listed is relevant to your study. If you are not sure if a list item applies to your research, read the appropriate section before selecting a response.

Materials & experimental systems

n/a	Involved in the study	
☐	☒	Antibodies
☒	☐	Eukaryotic cell lines
☒	☐	Palaeontology and archaeology
☐	☒	Animals and other organisms
☒	☐	Human research participants
☒	☐	Clinical data
☒	☐	Dual use research of concern

Methods

n/a	Involved in the study	
☒	☐	ChIP-seq
☐	☒	Flow cytometry
☒	☐	MRI-based neuroimaging

Antibodies

Antibodies used

Anti-murine adiponectin (rabbit) Immunodiagnostics, Hong Kong, China 12010
Anti-cytokeratin 5 (goat) Santa Cruz Biotechnology, Santa cruz, CA sc-17090
Anti-cytokeratin 8 (goat) Santa Cruz Biotechnology sc-241376
Anti-neuropilin-1 (goat) R&D system Inc., Minneapolis, MN, USA AF566
Anti-CD31 (goat) R&D system Inc., Minneapolis, MN, USA AF3628
Anti-galectin-3 (rat) Santa Cruz Biotechnology sc-23938
Anti-β5t (rabbit) MBL Life science, Nagoya, Japan PD021
Anti-β-actin (mouse) Sigma-Aldrich, St Louis, MI, USA A1978
Anti-neuropilin-1 (mouse) R&D, Minneapolis, MN, USA. AF566
Anti-CD72 (mouse) R&D, Minneapolis, MN, USA. AF1279
Anti-CD100 (mouse) Abcam, Cambridge, UK Ab231961
Anti-CD100 (human) LS-Bio, Seattle, WA, USA LS-B12098-50
Anti-mouse CD16/32 Purified eBioscience 14-0161-82
Pacific BlueTM anti-mouse CD3 Biolegend 100214
PE-CF594 anti-mouse CD4 BD HorizonTM 562285
Pacific BlueTM anti-mouse CD4 Biolegend 100428
Alexa Fluor® 647 anti-mouse CD8a Biolegend 100724
Pacific BlueTM anti-mouse CD4 Biolegend 100728
FITC anti-mouse CD44 BD PharmingenTM 553133
PE-CyTM7 anti-mouse CD25 BD PharmingenTM 552880
Pacific BlueTM anti-mouse CD25 Biolegend 102021
PE anti-mouse CD45 Biolegend 103106
Pacific BlueTM anti-mouse CD45 Biolegend 103125
FITC anti-mouse CD45 Biolegend 103107
APC anti-mouse CD326 (EpCAM) Biolegend 118214

PE/Cy7 anti-mouse Ly51 Biolegend 108314
Fluorescein Ulex Europaeus Agglutinin I (UEAI) Vector Laboratories, Inc. Burlingame, CA FL-1061
APC anti-mouse CD117 (c-kit) Biolegend 135108
PE anti-mouse CD24 BD PharmingenTM 553262
V450 mouse Lineage antibody cocktail BD 561301
Brilliant Violet 421TM anti-rabbit IgG Biolegend 406410

Validation

All results have been carefully validated by referring to the product manuals, literature publications, the relevant protein target information, or compared to other sources of the products.

Animals and other organisms

Policy information about studies involving animals; ARRIVE guidelines recommended for reporting animal research

Laboratory animals

C57BL/6J 000664 from Jackson Laboratory
B6;129-Adipoqtm1Chan/J 008195 from Jackson Laboratory
B6;FVB-Tg(Adipoq-cre)1Evdr/J 010803 from Jackson Laboratory
B6.129(Cg)-Gt(ROSA)26Sortm4(ACTB-tdTomato,-EGFP)Luo/J 007676 from Jackson Laboratory
FVB/N-Tg (MMTV-PyVT)634 Mul/J 002374 from Jackson Laboratory
NOD.CB17-Prkdcscid/J NOD/SCID; 001303 mice from the Jackson Laboratory

Wild animals

The study did not use wild animals.

Field-collected samples

The study did not use field-collected samples.

Ethics oversight

Animal usage and experiment were advised and approved by the Committee on the Use of Live Animals and Teaching and Research (CULATR) at The University of Hong Kong

Note that full information on the approval of the study protocol must also be provided in the manuscript.

Flow Cytometry

Plots

Confirm that:

☒ The axis labels state the marker and fluorochrome used (e.g. CD4-FITC).

☒ The axis scales are clearly visible. Include numbers along axes only for bottom left plot of group (a 'group' is an analysis of identical markers).

☒ All plots are contour plots with outliers or pseudocolor plots.

☒ A numerical value for number of cells or percentage (with statistics) is provided.

Methodology

Sample preparation

Preparation of thymic cell suspension
Freshly collected thymus was cut, minced and transferred to Dulbecco's Modified Eagle Medium (DMEM) containing 2 mg/ml collagenase Type I (GibicoTM, Waltham, MA, U.S.A.) and 40 µg/ml DNase I (Sigma-Aldrich, St. Louis, MO, U.S.A.). After incubation at 37°C with shaking for 30 minutes, cells were strained through a 100 µm filter mesh and centrifuged at 400 x g. The pellets were re-suspended in phosphate-buffered saline (PBS) and then labeled with specific antibodies for subsequent flow cytometric analyses. Where indicated, TNC complexes were enriched from enzyme-digested thymic cell suspensions by four-step 1 x g sedimentation in fetal bovine serum (FBS). Single cell suspension was obtained from the enriched TNC samples by gentle mechanical dissociation of the complexes with a 3 ml syringe and a 29 G needle.
Preparation of blood cell suspension
For peripheral blood analysis, EDTA was used as an anticoagulant reagent and added at a concentration of 1.5 mg per ml. The erythrocyte-lysis buffer (555899; BD Biosciences) was used to prepare blood samples for flow cytometric analysis and cell sorting. The mixed panel of BV421-conjugated anti-CD3, PE-CF594-conjugated anti-CD4 and Alexa Fluor 647-conjugated anti-CD8 was used to examine or sort T-helper, T-cytotoxic and immature CD4+CD8+ cells.
Preparation of lymphocyte suspension from liver
After perfusion with PBS through portal vein, liver tissues were dissected, homogenized and digested in DMEM containing 0.5 mg/ml collagenase Type IV (GibicoTM) and 150 µg/ml DNase I (Sigma-Aldrich) at 37°C for 40 minutes with shaking. After centrifugation at 1600 rpm for 5 minutes cell pellets were resuspended in PBS and went through a 100 µm cell strainer before loading onto a gradient containing 40% and 70% Percoll (GE Healthcare Bio-Sciences, Sweden). The fractionation was performed by centrifugation at 1126 x g for 20 minutes at 4°C. The middle layer containing lymphocytes was collected for subsequent analyses.
Preparation of stromal vascular fraction from adipose tissue
Epididymal adipose tissues were dissected, homogenized and digested in DMEM containing 1 mg/ml collagenase Type II (GibicoTM) and 150 µg/ml DNase I (Sigma-Aldrich) at 37°C for 30 minutes with shaking. After centrifugation at 1600 rpm for 10 min, cell pellets were resuspended in PBS and went through a 100µm cell strainer.

Instrument

Multicolor flow cytometry and cell sorting were performed with BD LSR Fortessa Analyzer (BD Bioscience, San Jose, CA, U.S.A.) and BD FACSAriaTM SORP Cell Sorter (BD Bioscience), respectively.

Software	BD FACSDiva™ (Version:6.1, BD), FlowJo (Version: 10.0.7, BD)
Cell population abundance	Purity of sorted cells was confirmed by repeated testing using the same Flow cytometry instrument with the same voltage and gating parameters.
Gating strategy	Dead events were excluded by FSC-A/SSC-A gating and adhesion events excluded by FSC-A/FSC-H gating.

☒ Tick this box to confirm that a figure exemplifying the gating strategy is provided in the Supplementary Information.
